# Effect of GnRH agonist alone or combined with different low-dose hCG on cumulative live birth rate for high responders in GnRH antagonist cycles: a retrospective study

**DOI:** 10.1186/s12884-022-04499-0

**Published:** 2022-03-02

**Authors:** Yuxia He, Yan Tang, Shiping Chen, Jianqiao Liu, Haiying Liu

**Affiliations:** 1 Department of Obstetrics and Gynecology, Center of Reproductive Medicine, Key Laboratory for Major Obstetric Diseases of Guangdong Province, The Third Affiliated Hospital of Guangzhou Medical University, Guangzhou, China; 2 Key Laboratory for Reproductive Medicine of Guangdong Province, The Third Affiliated Hospital of Guangzhou Medical University, Guangzhou, China; 3 Department of Obstetrics and Gynecology, Center of Reproductive Medicine, Zhongshan City People’s Hospital, Zhongshan, China; 4 Department of Reproductive Medicine, The Third Affiliated Hospital of Guangzhou Medical University, 63 Duobao Road, Guangzhou, Guangdong China

**Keywords:** GnRHa trigger, Low-dose hCG, Oocyte maturation, OHSS, Cumulative live birth rate

## Abstract

**Background:**

There is insufficient evidence regarding the impact of dual trigger on oocyte maturity and reproductive outcomes in high responders. Thus, we aimed to explore the effect of gonadotropin-releasing hormone agonist (GnRHa) trigger alone or combined with different low-dose human chorionic gonadotropin (hCG) regimens on rates of oocyte maturation and cumulative live birth in high responders who underwent a freeze-all strategy in GnRH antagonist cycles.

**Methods:**

A total of 1343 cycles were divided into three groups according to different trigger protocols: group A received GnRHa 0.2 mg (*n* = 577), group B received GnRHa 0.2 mg and hCG 1000 IU (*n* = 403), and group C received GnRHa 0.2 mg and hCG 2000 IU (*n* = 363).

**Results:**

There were no significant differences in age, body mass index, and rates of oocyte maturation, fertilization, available embryo, and top-quality embryo among the groups. However, the incidence of moderate to severe ovarian hyperstimulation syndrome (OHSS) was significantly different among the three groups (0% in group A, 1.49% in group B, and 1.38% in group C). For the first frozen embryo transfer (FET) cycle, there were no significant differences in the number of transferred embryos and rates of implantation, clinical pregnancy, live birth, and early miscarriage among the three groups. Additionally, the cumulative ongoing pregnancy rate and cumulative live birth rate were not significantly different among the three groups. Similarly, there were no significant differences in gestational age, birth weight, birth height, and the proportion of low birth weight among subgroups stratified by singleton or twin.

**Conclusions:**

GnRHa trigger combined with low-dose hCG (1000 IU or 2000 IU) did not improve oocyte maturity and embryo quality and was still associated with an increased risk of moderate to severe OHSS. Therefore, for high responders treated with the freeze-all strategy, the single GnRHa trigger is recommended for final oocyte maturation, which can prevent the occurrence of moderate to severe OHSS and obtain satisfactory pregnancy and neonatal outcomes in subsequent FET cycles.

## Background

In vitro fertilization-embryo transfer (IVF-ET) technology has earned its reputation as a major medical breakthrough in the twentieth century and has been applied to couples with tubal infertility, endometriosis, poor semen quality, and unexplained infertility [1]. The number of infertile couples who choose this technique to achieve conception has increased yearly. Controlled ovulation hyperstimulation (COH) plays an important role during IVF treatment, which aims to obtain an appropriate number of eggs and produce an appropriate number of embryos, thereby improving pregnancy outcomes. However, this technology is associated with several complications.

Ovarian hyperstimulation syndrome (OHSS) is one of the complications of COH, and it can increase the suffering of patients, result in additional medical expenses, and endanger the lives of patients in severe cases [2]. Studies have shown that gonadotropin-releasing hormone agonist (GnRHa) trigger can effectively induce final oocyte maturation and significantly reduce the incidence of OHSS in patients undergoing GnRH antagonist therapy [3–5]. However, for the fresh embryo transfer cycle, GnRHa trigger significantly reduces the occurrence of OHSS, but the clinical pregnancy rate is lower, and the miscarriage rate is higher than treated with human chorionic gonadotropin (hCG), which is a classic traditional trigger drug [6, 7]. This may be related to the low level and short duration of endogenous luteinizing hormone (LH) peak induced by GnRHa trigger, which results in defective corpus luteum function and impaired endometrial receptivity [8–10]. Therefore, some scholars have proposed a “dual trigger” strategy comprising GnRHa and low-dose hCG, which can strengthen luteal phase function and obtain comparable pregnancy outcomes to those treated with the hCG trigger [11–14]. However, the strategy of GnRHa trigger plus low-dose hCG is still associated with the risk of severe OHSS [15, 16]. Currently, the freeze-all policy is another important strategy to prevent OHSS for anticipated high responders [17]. Therefore, for patients who require a freeze-all strategy to prevent OHSS, it seems that there is no need to add low-dose hCG to GnRHa for final oocyte maturation due to the increased risk of OHSS, and the luteal phase insufficiency triggered by GnRHa is unimportant because it is unnecessary to achieve ongoing pregnancy. However, there are concerns that GnRHa trigger is not appropriate for all patients, and a small subset of patients have a suboptimal response to GnRHa trigger alone, which may result in a decreased oocyte yield, oocyte maturity, and available embryos, ultimately affecting pregnancy outcomes [18]. However, for oocyte donors suspected of normal and high response, GnRHa trigger maximizes mature oocyte yields but offers a significant reduction in OHSS risk compared to the dual trigger of GnRHa and hCG 2500 IU [19]. Therefore, there is insufficient evidence regarding the impact of the dual trigger on oocyte maturity and reproductive outcomes in high responders. Additionally, these studies did not focus on the cumulative live birth rate (CLBR), which is regarded as a significant indicator for assessing the success of assisted reproductive technology (ART). 

In response to the above problems, we retrospectively explored the effect of GnRHa trigger alone or combined with different low doses of hCG on oocyte maturity, embryo quality, OHSS incidence, pregnancy, and neonatal outcomes of subsequent frozen embryo transfer (FET) cycles for high responders undergoing the freeze-all strategy.

## Materials and methods

### Study population and grouping

This retrospective cohort study was conducted from March 2016 to December 2019 in the Department of Reproductive Medicine of the Third Affiliated Hospital, Guangzhou Medical University (Guangzhou, China). Infertile patients who received the GnRH antagonist regimen and final oocyte maturation triggered by GnRHa alone or combined with low-dose hCG were included. The inclusion criteria were as follows: (1) age ≤ 40 years old; (2) retrieved oocytes ≥15; and (3) freeze-all strategy. The exclusion criteria were as follows: (1) embryos derived from donated/vitrified oocytes; (2) preimplantation genetic testing cycles; (3) stage III-IV endometriosis/adenomyosis; (4) presence of uterine cavity lesions or anomalies; (5) untreated hydrosalpinx prior to FET; and (6) uncontrolled systemic diseases, such as hypertension, endocrine disorder, autoimmune disease and so on.

A total of 1343 cycles met inclusion criteria were divided into three groups according to different trigger protocols: group A received GnRHa 0.2 mg (*n* = 577), group B received GnRHa 0.2 mg combined with hCG 1000 IU (*n* = 403), and group C received GnRHa 0.2 mg combined with hCG 2000 IU (*n* = 363). The study protocol was approved by the institutional ethics committee of the Third Affiliated Hospital of Guangzhou Medical University.

### Ovarian stimulation, vitrification, and warming

All the patients in this study underwent a flexible GnRH antagonist protocol as previously described [20]. Briefly, the injection of follicle-stimulating hormone (FSH; Merck Serono, Geneva, Switzerland; Lizhu Group Co., Ltd., Zhuhai, China) were started from the second or third day of the menstrual cycle. The starting dose was adjusted according to follicular growth which were monitored based on the transvaginal ultrasound and serum hormone levels. The antagonist (Cetrorelix, Merck Serono, Darmstadt, Germany) at 0.25 mg/day was routinely started on the fifth or sixth day of ovarian stimulation. When at least three follicles reached or exceeded an average diameter of 17–18 mm, GnRHa 0.2 mg alone or combined with low-dose hCG (1000 IU or 2000 IU) were administered to trigger, and oocyte retrieval was scheduled 36–38 h later. Choosing GnRHa single trigger or dual trigger was based on physician preference. Conventional IVF or intracytoplasmic sperm injection (ICSI) was performed according to the sperm quality.

All available embryos were cryopreserved by vitrification method according to the manufacturer’s instruction (Kitazato Biopharma Co. Ltd. Shizuoka. Japan) as described previously [20]. On the morning of embryo transfer, embryo was warmed with a rapid thawing method. Thawed embryos were placed in a culture medium (K-SIBM, Cook) and cultured at 37 °C in a 6% CO_2_, 5% O_2,_ and 89% N_2_ incubator for about 4–5 h after thawing before embryo transfer.

### Endometrial preparation and embryo transfer

The selection of endometrial preparation was based on clinical assessment and patient preference. Endometrial preparation protocols, including the natural cycle (NC) and artificial programs, have been described previously [21]. Briefly, the NC protocol was applied to patients with regular menstrual cycles. The ovulation was determined by transvaginal ultrasonography monitoring and serum hormone levels, and the day of ovulation confirmation were considered day zero (P + 0). In the artificial protocol, daily oral estradiol valerate tablets (Progynova, Bayer, Germany) at 6 mg/day was started at day 2–4 of the menstrual cycle for at least 7 days and the dose was adjusted to 8-10 mg/day according to endometrial thickness and serum hormone levels. When the endometrial thickness reached ≥7 mm, vaginal progesterone (Crinone, Merck Serono, Germany) 90 mg once daily and oral progesterone (Duphaston, Abbott Biologicals B.V., Netherlands) 10 mg twice daily were provided for luteal support. The start day of progesterone exposure were considered day zero (P + 0).

One or two thawed embryos were transferred under ultrasound guidance using a soft-tipped Wallace (PortexLed., Hythe, United Kingdom) catheter on the fourth (cleavage-stage embryo, P + 3) or sixth (blastocyst, P + 5) day after ovulation confirmation or progesterone supplementation. Progesterone for luteal support after embryo transfer were used in all patients and continued taking until 14 days after FET, and maintained up to 10 weeks of gestational age in case of a pregnancy established.

### Outcome parameters

The primary outcomes were CLBR and the incidence of moderate to severe OHSS. Secondary outcomes included rates of oocyte maturation, available embryos, top-quality embryos, and neonatal outcomes. Neonatal outcomes included preterm birth, birth weight, birth height, and low birth weight.

The diagnosis and stage of OHSS were based on the criteria described by Golan et al. [22]. Moderate OHSS was defined as the presence of ascites on ultrasonography accompanied by abdominal distension and discomfort with or without nausea, vomiting, and/or diarrhea. Severe OHSS was diagnosed when there was clinical evidence of ascites and/or hydrothorax or breathing difficulties with or without hemoconcentration, coagulation abnormalities, and diminished renal function. Clinical pregnancy was confirmed by the observation of an intrauterine/extrauterine gestational sac diagnosed by ultrasound about 4 weeks after FET. Ongoing pregnancy was defined as a viable pregnancy progressing longer than 12 weeks of gestation. Live birth was defined as the delivery of an infant after 28 weeks of gestation with postnatal evidence of life. Cumulative live birth was defined as delivery that occurred during subsequent FET cycles after the freeze-all strategy from the same ovarian stimulation cycle, until a live foetus occurred or all embryos were used. Preterm birth was defined as the delivery of a live infant before 37 completed weeks of gestational age. Low birth weight was defined as the birth weight of newborn < 2500 g.

### Sample size

The sample size calculation was performed for primary outcomes of the study, the CLBR and the incidence of moderate to severe OHSS. For the calculation of the sample size of the CLBR, according to previous observations of Griesinger et al. [23], we calculated that at least 239 subjects per group were required to achieve 90% power for a two-sided Chi square test with an alpha of 0.05, a number that would increase to 266 to allow for a dropout rate of 10%. For the calculation of the sample size of the OHSS, based on the work of O’Neil et al. [15], we calculated that a sample size of 140 subjects in each group will provide 90% power to detect a significant difference at a significance level of 0.05. Assuming a 10% rate of withdrawal, it was planned to enroll 156 subjects. The number of each group of patients that met the inclusion criteria in this study exceeded the calculated sample size, indicating that the results of this study are reliable.

### Statistical analysis

Statistical analysis was performed using the Statistical Package for Social Science (SPSS, version 25.0, IBM, Armonk, NY, USA). Continuous variables were expressed as mean ± standard deviation (SD), and comparisons among groups were analyzed by the one-way analysis of variance. Categorical variables, described as frequencies and percentages, were compared by the chi-square test or Fisher’s exact test, as appropriate. *P* < 0.05 was considered statistically significant.

## Results

A total of 1343 IVF/ICSI cycles that met the study inclusion criteria were included in the analysis and divided into three groups according to different trigger protocols. There were 577 cycles in group A (GnRHa 0.2 mg), 365 cycles in group B (GnRHa 0.2 mg plus 1000 IU hCG), and 730 cycles in group C (GnRHa 0.2 mg plus 2000 IU hCG). The COH treatment characteristics, pregnancy outcomes, and neonatal outcomes were compared among the three groups.

The baseline and treatment characteristics of the patients stratified by the trigger method are shown in Table 1. There were no significant differences in terms of patient age, body mass index (BMI), infertility duration, antimüllerian hormone (AMH), basal FSH, duration of stimulation, total dose of gonadotropin, serum hormone levels on trigger day, number of retrieved oocytes and rates of oocyte maturation, fertilization, cleavage, two pronuclei, available day 3 embryo, top-quality day 3 embryo, and unavailable embryo cycles among the three groups. However, there was a significant difference among the three groups in terms of the incidence of moderate to severe OHSS (0% in group A, 1.49% in group B, and 1.38% in group C, Table 1).

**Table 1:** Baseline and treatment characteristics of patients stratified by the different trigger methods

	Group A: 0.2 mg GnRHa	Group B: 0.2 mg GnRHa+ 1000 IU hCG	Group C: 0.2 mg GnRHa + 2000 IU hCG	*p*
Cycles (n)	577	403	363	
Female age (year)	29.63 ± 3.61	29.78 ± 3.65	30.06 ± .379	0.226
BMI (kg/m^2^)	21.92 ± 3.54	22.16 ± 3.64	22.39 ± 3.47	0.136
Duration of infertility (years)	4.03 ± 2.59	4.19 ± 2.50	4.19 ± 2.54	0.509
AMH (ng/ml)	9.70 ± 3.76	9.52 ± 4.11	9.31 ± 4.40	0.343
Basal FSH (IU/L)	5.42 ± 2.28	5.49 ± 2.26	5.45 ± 2.27	0.347
Previous IVF/ICSI cycles				0.970
0	87.0 (502/577)	85.36 (344/403)	86.78 (315/363)	
1	10.57 (61/577)	11.41 (46/403)	10.19 (37/363)	
2	2.08 (12/577)	2.48 (10/403)	2.48 (9/363)	
≥ 3	0.35 (2/577)	0.75 (3/403)	0.55 (2/363)	
Type of infertility				0.799
Primary infertility	57.71 (333/577)	57.82 (233/403)	59.78 (217/363)	
Secondary infertility	42.29 (244/577)	42.18 (170/403)	40.22 (146/363)	
Duration of stimulation (days)	9.73 ± 1.68	9.73 ± 1.95	9.63 ± 1.63	0.656
Total dose of Gn (IU)	1539.37 ± 583.89	1541.69 ± 599.37	1561.19 ± 584.42	0.548
E_2_ Levels at trigger day (pmol/ml)	16,896.28 ± 3052.42	16,797.20 ± 2944.31	16,725.54 ± 3094.00	0.608
LH Levels at trigger day (IU/L)	1.79 ± 1.35	1.69 ± 1.35	1.71 ± 1.44	0.529
P Levels at trigger day (nmol/ml)	3.50 ± 1.68	3.46 ± 1.72	3.42 ± 1.69	0.798
No. of oocytes retrieved	22.93 ± 5.40	22.41 ± 5.25	22.69 ± 5.93	0.346
Oocyte maturation rate (%)	82.92 (1850/2231)	81.42 (1030/1265)	81.21 (869/1070)	0.369
Fertilization rate (%)	79.10 (10,157/12841)	79.0 (6946/8792)	78.78 (6323/8026)	0.860
Cleavage rate (%)	98.62 (10,017/10157)	98.23 (6823/6946)	98.48 (6227/6323)	0.121
2PN rate (%)	62.46 (8021/12841)	62.28 (5476/8792)	63.14 (5068/8026)	0.474
Available day 3 embryo rate (%)	56.07 (5617/10017)	57.01 (3890/6823)	56.50 (3518/6227)	0.483
Top-quality day 3 embryos rate (%)	20.33 (2036/10017)	20.75 (1416/6823)	20.06 (1249/6227)	0.606
Moderate to severe OHSS rate (%)	0 (0/577)	1.49 (6/403)	1.38 (5/363)	0.002
Unavailable embryo rate (%)	1.73 (10/577)	2.73 (11/403)	2.20 (8/363)	0.571

Comparisons of pregnancy outcomes of the first FET cycle and CLBR after the freeze-all strategy among the three groups are presented in Table 2. As of July 31, 2021, 16 patients had not undergone the FET cycle after the freeze-all strategy (ten cycles in group A, three cycles in group B, and three cycles in group C). The first FET was implemented in 1298 cycles after the freeze-all strategy as follows: 557 cycles in group A, 389 cycles in group B, and 352 cycles in group C. There were no significant differences in the number of transferred embryos, endometrial thickness, and rates of implantation, clinical pregnancy, early miscarriage, and live birth among the three groups. As of July 31, 2021, 33 patients had ongoing pregnancies, eight pregnant patients had been lost to follow-up, and 147 patients had undergone embryo transfer without live birth and had no remaining embryos. Embryo transfer was performed in 123 patients without live births but with remnant embryos. Ultimately, 987 patients achieved live births. The results showed that the cumulative ongoing pregnancy rate (80.24% in group A, 81.14% in group B, and 75.76% in group C) and CLBR (74.35% in group A, 75.68% in group B, and 69.70% in group C) were not significantly different among the three groups (Table 2).

**Table 2:** Pregnancy outcomes of the first FET cycle and cumulative reproductive outcomes in the study groups

	Group A: 0.2 mg GnRHa	Group B: 0.2 mg GnRHa+ 1000 IU HCG	Group C: 0.2 mg GnRHa + 2000 IU HCG	*P*
Cycles (n)	557	389	352	
No. of embryos transferred	1.53 ± 0.49	1.51 ± 0.50	1.59 ± 0.49	0.501
Embryo developmental stage at transfer				0.919
Day 3	17.06 (95/557)	18.77 (73/389)	20.45 (72/352)	
Day 4	0.54 (3/557)	0.77 (3/389)	1.14 (4/352)	
Day 5	70.74 (394/557)	69.41 (270/389)	68.47 (241/352)	
Day5 + Day 6	1.08 (6/557)	1.03 (4/389)	1.14 (4/352)	
Day6	10.59 (59/557)	10.03 (39/389)	8.81 (31/352)	
Endometrial thickness (mm)	8.64 ± 1.52	8.75 ± 1.52	8.72 ± 1.61	0.528
Pregnancy rate (%)	65.35 (364/557)	63.05 (247/389)	65.91 (232/352)	0.762
Implantation rate (%)	53.17 (453/852)	52.73 (309/586)	53.67 (300/559)	0.951
Early miscarriage rate (%)	10.16 (37/364)	9.31 (23/247)	12.07 (28/232)	0.599
Ectopic pregnancy rate (%)	1.65 (6/364)	1.62 (4/247)	1.72 (4/232)	0.996
Live birth rate (%)	54.22 (302/557)	54.50 (212/389)	54.26 (191/352)	0.996
Cumulative ongoing pregnancy rate (%)	80.24 (463/577)	81.14 (327/403)	75.76 (275/363)	0.141
Cumulative live birth rate (%)	74.35 (429/577)	75.68 (305/403)	69.70 (253/363)	0.143

Comparisons of neonatal outcomes of patients among the three groups stratified by singleton or twin birth are shown in Table 3. As of July 31, 2021, 429 patients in group A, 305 in group B, and 253 in group C had achieved a live birth. When patients were subgrouped by singleton or twin birth, there were no significant differences in terms of gestational age, birth weight, birth height, and rates of congenital anomalies and low-birth-weight infants among the three groups (Table 3).

**Table 3:** Neonatal outcomes of patients stratified by the different trigger methods

	Group A: 0.2 mg GnRHa	Group B: 0.2 mg GnRHa+ 1000 IU HCG	Group C: 0.2 mg GnRHa + 2000 IU HCG	*P*
Cycles (n)	429	305	253	
Preterm birth (<37 weeks)	22.84 (98/429)	20.98 (64/305)	19.76 (50/253)	0.619
Singleton	7.69 (33/429)	7.54 (23/305)	8.30 (21/253)	0.940
Twin	15.15 (65/429)	13.44 (41/305)	11.46 (29/253)	0.396
Gestational age (weeks)	37.30 ± 2.50	37.35 ± 2.38	37.57 ± 2.15	0.284
Singleton	38.39 ± 1.92	38.37 ± 1.68	38.33 ± 1.97	0.932
Twin	35.67 ± 2.09	35.48 ± 2.14	36.15 ± 1.70	0.272
Birth height (mm)	48.75 ± 2.68	48.35 ± 2.96	48.82 ± 2.52	0.046
Singleton	49.69 ± 2.09	49.46 ± 1.98	49.73 ± 2.22	0.313
Twin	46.89 ± 2.74	46.21 ± 3.34	47.00 ± 2.08	0.060
Birth weight (kg)	2955.06 ± 634.54	2892.02 ± 625.58	2961.52 ± 613.56	0.247
Singleton	3229.80 ± 535.51	3156.45 ± 491.02	3204.80 ± 557.60	0.255
Twin	2415.06 ± 436.87	2403.85 ± 550.01	2504.81 ± 425.31	0.205
Congenital anomalies rate (%)	0.58 (3/516)	0.27 (1/370)	0.33 (1/306)	0.747
Low birth weight rate (<2500 g)	22.67 (117/516)	23.51 (87/370)	21.24 (65/306)	0.779
Singleton	4.07 (21/516)	5.67 (21/370)	4.25 (13/306)	0.500
Twin	18.60 (96/516)	17.84 (66/370)	16.99 (52/306)	0.842

## Discussion

The results of this study demonstrate that the dual trigger comprising GnRHa and low-dose hCG (1000 IU or 2000 IU) for final oocyte maturation was associated with a significantly increased risk of moderate to severe OHSS compared to GnRHa trigger alone in high responders who underwent the freeze-all strategy. Moreover, we showed that the rates of oocyte maturation, fertilization, top-quality day 3 embryos, CLBR, and neonatal outcomes were comparable between the dual trigger and GnRHa single trigger. These results are crucial for guiding clinical practice and encouraging the use of GnRHa trigger alone in high responders in order to prevent the occurrence of moderate to severe OHSS and have no negative effects on pregnancy and neonatal outcomes for subsequent FET cycles.

OHSS is one of the serious iatrogenic complications in patients undergoing ART and is characterized by increased vascular permeability, hemoconcentration, and fluid leakage to the third space, which can cause liver and/or kidney damage, thrombosis, and life-threatening events in severe cases [16]. A variety of strategies have been developed to prevent and reduce the occurrence of OHSS, the most effective being the use of GnRHa instead of the traditional hCG to trigger final oocyte maturation in GnRH antagonist cycles [24, 25]. Engmann et al. showed that the GnRHa trigger alone could be used to completely prevent the occurrence of OHSS in the fresh embryo transfer cycle in high responders [4]. Another study also found that for patients with polycystic ovary syndrome (PCOS) treated with GnRH antagonist protocol, the most effective strategy to eliminate the incidence of moderate and severe OHSS was the GnRHa trigger for final oocyte maturation [26]. The results of the present study are consistent with these conclusions. For high responders, the strategy of GnRHa trigger alone combined with the freeze-all strategy can fully prevent the occurrence of moderate to severe OHSS.

The amplitude of the LH peak induced by GnRHa is smaller, and the duration is shorter, which may help to reduce the risk of OHSS [8, 27]. However, this is associated with insufficient corpus luteum function and decreased endometrial receptivity [9, 10, 28]. For the fresh embryo transfer cycle, the GnRHa trigger reduces the rates of embryo implantation, clinical pregnancy, and live birth and increases the early miscarriage rate compared with the hCG trigger [29]. Elective cryopreservation of all embryos after GnRHa trigger and transfer in subsequent FET cycles maintains an excellent pregnancy rate [23, 30]. Therefore, considering the impaired endometrial receptivity and to prevent the occurrence of moderate to severe OHSS, anticipated high responders in our study who received the GnRHa trigger alone canceled fresh cycle transfer and transferred in subsequent FET cycles.

The LH and FSH peaks produced by the GnRHa trigger are closer to the physiological condition, and combined with long half-life hCG, could improve luteal function and pregnancy outcomes in the fresh embryo transfer cycle compared to GnRHa trigger alone [31]. However, for patients who adopt the freeze-all strategy to prevent OHSS, it is unclear whether it is necessary to add a small dose of hCG because enhanced corpus luteal function is not required in these patients, and there is a potentially increased risk of moderate to severe OHSS compared to GnRHa trigger alone. Neill et al. [15] showed that GnRHa trigger plus low-dose hCG 1000 IU can significantly increase the incidence of moderate to severe OHSS in the fresh embryo transfer cycle compared to GnRHa alone (6.0% vs. 0%), and there were no significant differences in the rates of clinical pregnancy and spontaneous miscarriage. Severe early OHSS requiring ascites drainage and hospitalization can occur even after combined GnRHa trigger and 1500 IU hCG for luteal rescue [16]. Moreover, Jones et al. found that, compared with GnRHa single trigger in donated oocyte cycles, GnRHa trigger plus low-dose hCG 2000 IU was associated with a significantly increased incidence of OHSS (8.5% vs. 0.4%) [17]. However, Shapiro et al. revealed that the ongoing pregnancy rate was significantly increased with the dual trigger, whereas the incidence of OHSS was comparable to that of GnRHa alone [11]. Daniel et al. also showed that the dual trigger improved the probability of conception and live birth without increasing the risk of significant OHSS in high responders [31]. The important reason for the inconsistency of conclusions may be due to the heterogeneity of the infertile population, small sample sizes, and different dosages of hCG. Our results suggest that, for high responders that undergo the freeze-all strategy, one disadvantage of using low-dose hCG with GnRH trigger is the risk of moderate to severe OHSS; thus, it may not be necessary to add a small dose of hCG for them.

Clinicians hesitate to use GnRHa trigger alone, mainly because some patients may not be able to produce sufficient LH levels because of their poor response to the GnRHa trigger, which affects the final oocyte maturation and may ultimately result in poor pregnancy outcomes [18]. A study suggested that a dual trigger using GnRHa and low-dose hCG may be associated with a modest increase in oocyte yield, both in terms of number and maturity, compared to GnRHa single trigger [15]. However, Jones et al. found that the dual trigger of GnRHa plus 2000 IU hCG did not improve the oocyte maturation rate compared with GnRHa single trigger for donated oocyte cycles [19]. In addition, another study showed that the addition of hCG (1500–5000 IU) to GnRHa trigger did not improve the oocyte maturation rate in normal and low responders undergoing planned oocyte cryopreservation, and it suggested that GnRHa trigger alone is an appropriate choice for those patients regardless of the risk of OHSS [32]. In this study, we expanded the sample size to better explore the effect of GnRHa trigger combined with different doses of hCG on oocyte maturation rate and embryo quality. The results showed that GnRHa plus low-dose hCG (1000 IU or 2000 IU) did not significantly improve the rates of oocyte maturation, available embryos, and top-quality embryos. Most importantly, there was no significant difference in CLBR. However, there was a significantly increased risk of moderate to severe OHSS. Therefore, our study suggests that, for high responders treated with the freeze-all strategy using the GnRH antagonist program, GnRHa single trigger does not affect the oocyte maturity and could prevent the occurrence of moderate to severe OHSS compared to the dual trigger. Moreover, the GnRHa trigger can also achieve satisfactory pregnancy outcomes in subsequent FET cycles.

To the best of our knowledge, no study has explored the effect of GnRHa plus low-dose hCG trigger on child safety. These results, for the first time, showed no significant difference in gestational age, birth height and weight, and low birth weight rate among the three groups stratified by singleton or twin, which suggested that the addition of low-dose hCG (1000 or 2000 IU) did not significantly improve embryo quality. Additionally, there was no significant difference in the CLBR during the subsequent FET cycles after whole embryo freezing among the three groups. This information seems important as it could encourage clinicians to use GnRHa trigger alone for the final oocyte maturation without concerns whether this strategy has an effect on embryo quality and neonatal outcomes for high-response patients.

For patients with good ovarian reserve, the administration of high doses of FSH may induce an excessive ovarian response, resulting in a high risk of OHSS. An individualized controlled ovarian stimulation (COS) protocol with an appropriate starting dose of FSH can reduce OHSS risks and financial costs [33]. To date, fewer studies have proposed some models with several biomarkers of ovarian reserve as a means for selecting the FSH starting dose to obtain optimal ovarian response [33, 34]. A nomogram suggested by La Marca based on age, FSH, and AMH, is associated with an increase in the proportion of patients with an optimal response [34]. Similarly, another predictor index proposed by Oliveira et al., the ovarian response prediction index (ORPI), which is calculated by multiplying the AMH level by the antral follicle count and divided by the patient’s age, is excellent in predicting an excessive response with a predictive capability higher than each marker individually [33]. Moreover, the results of Carla Peluso et al. confirmed the above-mentioned findings and showed that the ORPI was the strongest predictor of hyper-response in normovulatory infertile women [35]. These prediction models mentioned in these studies are used for patients with normal regular menstrual cycles. Nevertheless, current available evidence regarding PCOS women, particularly those with high AMH and BMI, does not seem adequate. Papler et al. [36] showed that obese women require significantly larger amounts of gonadotropins to achieve similar ovarian stimulation as normal weighting women. Therefore, when calculating the starting dose of FSH for PCOS, especially in obese women, the patients’ weight should be taken into consideration to achieve a satisfactory ovarian response.

At present, reproductive specialists still face great challenges for some infertile patients affected by particular conditions, such as endometriosis and PCOS. One retrospective cohort study found a significantly longer time to pregnancy and lower pregnancy rate and live birth rate in the women with diagnostic laparoscopy only compared to women whose visible endometriosis is completely removed using laparoscopy prior to ART [37]. However, laparoscopic excision of endometriotic ovarian cysts is associated with a significant reduction in ovarian reserve [38]. Therefore, whether surgery should be performed prior to ART to improve reproductive outcomes is still controversial. Individualized treatment is needed in selecting appropriate treatment strategies for different groups of people. For young patients with good ovarian reserve, IVF should be reserved as the secondary treatment for women who fail to conceive spontaneously after surgery within 6–12 months [39]. For another special population, PCOS patients, the above-mentioned individualized COS and the GnRHa single trigger discussed in this study could reduce the OHSS risks. In addition, Fabio et al. showed that the number of mature oocytes and pregnancy rate in PCOS patients treated with oral myo-inositol (MI), an insulin-sensitizing agent, were significantly higher than that of no treatment control. Moreover, MI therapy can reduce the total amount of gonadotropin, the duration of COS, and the overall cost of IVF procedures [40]. Therefore, for potentially high-responders, such as PCOS women, the strategy of MI supplementation combined with GnRHa single trigger may be an effective method to combat OHSS. Further studies will be necessary to evaluate the possible applicability of this strategy.

This study has some limitations. First, it only included high responders, making the conclusions of this study unsuitable for patients with low and normal responses. In addition, the results of the study may be biased because of its retrospective nature; therefore, prospective studies are required to verify the conclusions of this study. However, to the best of our knowledge, the number of patients included in this study for each group was larger than that of other similar studies, making the results of this study valuable for confidently guiding the use of GnRHa trigger alone for high responders without the addition of low-dose hCG.

## Conclusions

Our results showed that, compared with GnRHa trigger alone, GnRHa combined with low-dose hCG (1000 or 2000 IU) failed to improve the oocyte maturity and embryo quality in high responders undergoing GnRH antagonist regimen but was associated with an increased risk of moderate to severe OHSS. Therefore, the GnRHa single trigger is recommended to induce final oocyte maturation in high responders undergoing a freeze-all strategy, which can prevent the occurrence of moderate to severe OHSS and obtain satisfactory pregnancy and neonatal outcomes during subsequent FET cycles.

## Data Availability

The datasets used and/or analyzed during the current study are available from the corresponding author upon reasonable request.

## Abbreviations

AMH: Antimüllerian hormone
ART: Assisted reproductive technology
BMI: Body mass index
CLBR: Cumulative live birth rate
COH: Controlled ovulation hyperstimulation
COS: Controlled ovarian stimulation
FET: Frozen embryo transfer
FSH: Follicle-stimulating hormone
GnRHa: Gonadotropin-releasing hormone agonist
hCG: Human chorionic gonadotropin
ICSI: Intracytoplasmic sperm injection
IVF: In vitro fertilization
LH: luteinizing hormone
OHSS: Ovarian hyperstimulation syndrome
PCOS: Polycystic ovarian syndrome
